# Metabolomic Profiling and In Vitro Evaluation of Cytotoxic, Genotoxic, and Antigenotoxic Effects of *Staphylea pinnata* L. Extract from Italian Flora

**DOI:** 10.3390/biom15030385

**Published:** 2025-03-06

**Authors:** Ghanya Al-Naqeb, Fabio Pietrolucci, Mauro Commisso, Aliki Kalmpourtzidou, Amanda Oldani, Sara Boussetta, Beatrice Maccarini, Rachele De Giuseppe, Hellas Cena

**Affiliations:** 1 Laboratory of Dietetics and Clinical Nutrition, Department of Public Health, Experimental and Forensic Medicine, University of Pavia, 27100 Pavia, Italy; aliki.kalmpourtzidou01@universitadipavia.it (A.K.); sara.boussetta01@universitadipavia.it (S.B.); beatrice.maccarini01@universitadipavia.it (B.M.); rachele.degiuseppe@unipv.it (R.D.G.); hellas.cena@unipv.it (H.C.); 2 Department of Food Sciences and Nutrition, Faculty of Agriculture Food and Environment, University of Sana’a, Sana’a P.O. Box 1247, Yemen; 3 Department of Biotechnology, University of Verona, 37134 Verona, Italy; fabio.pietrolucci@univr.it (F.P.); mauro.commisso@univr.it (M.C.); 4 PASS-Bio Med, Centro Grandi Strumenti, University of Pavia, 27100 Pavia, Italy; amanda.oldani@unipv.it; 5 Clinical Nutrition Unit, ICS Maugeri IRCCS, 27100 Pavia, Italy

**Keywords:** *Staphylea pinnata* L., metabolomic profile, Italian flora, cytotoxicity, genotoxicity

## Abstract

*Staphylea pinnata* L., (*S. pinnata*), has long been recognized in Europe as both a wild food source and a traditional medicinal. This study aimed to characterize the metabolomic profile of the leaf extract of *S. pinnata* and assess its cytotoxic, genotoxic, and antigenotoxic effects in vitro for the first time. The methanolic extract of the leaves was analyzed using Ultra-Performance Liquid Chromatography–High-Resolution Mass Spectrometry (UPLC-HRMS). To evaluate its cytotoxic, genotoxic, and antigenotoxic properties, the cytokinesis block micronucleus assay was performed on Chinese hamster ovarian K1 cells. The analysis revealed a wide variety of metabolites in the extract, with B-type procyanidins and prodelphinidins being the most abundant. The genotoxicity of the extract varied depending on its concentration; at the lowest concentration (75 μg/mL), it showed no genotoxic effects and exhibited antigenotoxic properties by reducing the frequency of micronuclei induced by mitomycin C. However, at the highest concentration (300 μg/mL), the extract demonstrated genotoxic effects. In conclusion, the *S. pinnata* extract displayed both genotoxic and antigenotoxic properties, which may be attributed to its phytochemical composition. These findings highlight the complex nature of the plant’s bioactive compounds, suggesting potential therapeutic applications with careful consideration of dosage. Additional research is necessary to understand the mechanisms underlying these properties.

## 1. Introduction

The only surviving species of the *Staphylea* genus that is indigenous to Central and Eastern Europe is *Staphylea pinnata* L., (*S. pinnata*), also referred to as the European bladdernut. It is a member of the family Staphyleaceae [1]. This species of plant is found throughout Italy [2]. Historically, *S. pinnata* has served as a wild food source in Europe, with its seeds and flowers being used in teas and pickling [2,3]. Additionally, its spring buds have been pickled or consumed as a side dish [3]. The roasted seeds of *S. pinnata* are also used in the production of liquor and schnapps [4]. The seed kernels of *S. pinnata* have shown to be a valuable source of many bioactive components including α-tocopherol, chlorophyll, lutein, and n-6 to n-3 fatty acids [5]. Research also suggests that *S. pinnata* seeds can be used as a source of edible oil [3]. Some studies reported the use of *S. pinnata* as a traditional medicine [6]. Extract from flowers and leaves of *S. pinnata* has shown different biological activities including, antioxidants [7], cytotoxic, and antibacterial properties [8].

Plant extracts and their constituents are widely regarded as valuable sources of bioactive compounds, in high demand across various industries, including food and pharmaceuticals [9]. However, their potential to induce genotoxic effects, combined with a lack of sufficient research into their beneficial properties, significantly limits their use [10]. While herbal medicines are often considered safe due to their long history of use, it is important to recognize that some plants utilized in traditional medicine have demonstrated genotoxicity [11,12]. The genotoxic or antigenotoxic effects of most plant extracts used in traditional medicine remain uncertain.

Some *S. pinnata* research focused on specialized metabolites such as polyphenols, flavonoids, and hydroxycinnamic derivatives, which may have antibacterial, antiproliferative, and antioxidant properties [13]. A previous study identified phytochemicals like quercetin and isorhamnetin glycosides in the hydroalcoholic extract of the aerial parts, flowers, and leaves of the *S. pinnata* plant [1]. Isoquercetin and quercetin malonyl glucoside exhibited potent antioxidant, antimicrobial, and wound healing properties, making them valuable as antiaging ingredients for cosmeceutical and therapeutic applications to heal skin wounds [1].

Quercetin and isorhamnetin were reported to reduce benzo[a]pyrene-induced genotoxicity [14]. Additionally, it has been reported that low doses of quercetin can reduce cytogenetic damage caused by mitomycin C (MMC) and hydrogen peroxide [15,16]. In contrast, it was observed that quercetin and its natural glycoside isoquercetin exhibited cytotoxicity and genotoxicity by inducing genetic instability and increasing micronuclei formation in the Chinese hamster ovary K1 (CHO-K1) rodent mammalian cell line [17]. The presence of these active metabolomics in *S. pinnata* extracts even as a minor constituent may contribute to its genotoxic or antigenotoxic potential. Information regarding the potential genotoxicity of *S. pinnata* is limited in the literature as the plant is mainly recognized for its decorative and intermittent culinary purposes rather than its medicinal or toxicological characteristics. Nevertheless, it is crucial to explore the genotoxicity and antigenotoxicity of this plant extract to guarantee its safety, since it is used in traditional medicine. The primary aim of this study was to characterize the metabolomic profile of the methanolic extract from *S. pinnata* leaves using Ultra-Performance Liquid Chromatography–High-Resolution Mass Spectrometry (UPLC-HRMS). Additionally, we assessed its cytotoxic, genotoxic, and antigenotoxic effects in CHO-K1 cells through the cytokinesis block micronucleus (CBMN) assay. The automated in vitro CBMN assay was performed using an image analysis approach, with analysis carried out via a widefield fluorescence microscope.

This study is conducted under the auspices of the Italian National Biodiversity Center. Our focus is on assessing the genotoxic and antigenotoxic properties of medicinal plants native to Italy, with *S. pinnata* being one of the species examined. A significant aspect of this research is the inaugural evaluation of the genotoxicity and antigenotoxicity of *S. pinnata*. The findings from this study will yield important preliminary information regarding the safety profile of this plant.

## 2. Materials and Methods

### 2.1. Chemicals

Cell culture media, supplements, consumables, dimethyl sulfoxide (DMSO; 99.9%), and formaldehyde solution were all acquired from Euroclone S.p.A. (Milan, Italy). Methanol (HPLC grade) was supplied by Merck KGaA (Darmstadt, Germany). Mitomycin C and cytochalasin B were obtained from D.B.A. Italia s.r.l. (Milan, Italy), while 3-(4,5-dimethylthiazol-2-yl) 2,5-diphenyltetrazolium bromide (MTT) was sourced from Biosigma S.p.A. (Cona, Italy). Hoechst 33342 Staining Dye Solution from Abcam, supplied by Prodotti Gianni s.r.l. (Milano, Italy).

### 2.2. Sample Collections and Extraction

Three different pools of leaves of *S. pinnata* were collected from plants cultivated at the botanical garden of Padua (Italy) in the full vegetative phase on 28 September 2022. Each pool included leaves picked up by two individuals. The samples were promptly frozen in liquid nitrogen and then ground into a fine powder using an A11 basic analytical mill (IKA-Werke, Staufen, Germany). They were subsequently stored at −80 °C in plastic centrifuge tubes wrapped in foil.

### 2.3. Preparation of S. pinnata Leaves Methanolic Extract

Prior to the chemical and biological activities, plant extracts were prepared as follows. About 1 g of frozen powder of *S. pinnata* leaves was extracted with 10 volumes (*w*/*v*) of 100% LC-MS grade methanol (Honeywell, Seelze, Germany). The samples underwent vortexing for 30 s, followed by sonication on ice for 10 min with a 40 kHz ultrasonic bath (SOL-TEC, Milano, Italy). Subsequently, samples were centrifuged at 14,000× *g* for 10 min at a temperature of 4 °C. The supernatants were then collected and preserved at −20 °C. For the assessment of genotoxicity, 20 g of frozen *S. pinnata* leaf powder was subjected to extraction with methanol at a 1:10 (*w*/*v*) ratio in sealed glass containers. The mixture was agitated using a magnetic stirrer for 48 h at an ambient temperature. After this period, the methanolic extracts were filtered through quantitative filter paper (ArtiGloss, with a particle retention range of 12–15 μm) and concentrated using rotary evaporation (BUCHI R-210) at 40 °C for 30 to 50 min. To ensure complete removal of methanol, the extract was placed in a fume hood for 24 h and subsequently freeze-dried with liquid nitrogen. The final extract was then transferred to amber glass bottles and stored at 4 °C for future analysis. The yield of the extract was calculated using the appropriate formula as outlined in Equation (1):

(1) $$Yield\;\%=\frac{weight\;of\;obtained\;extract(g)}{weight\;of\;dried\;plant\;sample\;used(g)}\times 100.$$

This method resulted in a final yield of 2% for the methanolic extract of *S. pinnata*. The concentrated extracts were then placed into small glass tubes and stored at a temperature of 4 °C for future analyses.

### 2.4. Metabolomic Profile of S. pinnata Methanolic Extract

The hydro-alcoholic extracts were first diluted 1:10 with LC-MS grade water (Honeywell) and subsequently filtered through 0.22 μm Minisart filters (Sartorius-Stedim Biotech, Gottingen, Germany). The metabolomic profile was then obtained using Ultra-Performance Liquid Chromatography coupled with High-Resolution Mass Spectrometry (UPLC-HRMS) (Waters, Manchester, UK). The instrumental configuration and analytical procedures were adapted from a previously published protocol, with modifications [18]. Briefly, an ACQUITY I CLASS Ultra Performance Liquid Chromatography (UPLC) system, coupled with an ACQUITY Photodiode Array (PDA) detector, was interfaced with an electrospray ionization (ESI) source and a Xevo G2-XS quadrupole time-of-flight (qTOF) mass spectrometer (Waters, Manchester, UK). The PDA detector utilized an eλ detector with a spectral range of 190–800 nm and a high-sensitivity flow cell. Chromatographic separation was achieved using a Waters BEH C18 reversed-phase column (2.1 mm × 100 mm, 1.7 µm), coupled to a Vanguard pre-column (2.1 mm × 5 mm, 1.7 µm), with the column compartment maintained at 30 °C. The mobile phase consisted of solvent A (0.1% formic acid in LC-MS grade water) and solvent B (100% acetonitrile), with a flow rate set at 0.350 mL/min. The gradient elution started at 1% B, held for 1 min, and then increased linearly to 40% B at 10 min, 70% B at 13.5 min, 90% B at 15 min, and 99% B at 16.5 min. The gradient was then maintained at 99% B for 3.5 min before decreasing to 1% B at 20.1 min. The system remained in an isocratic mode (1% B) for the final 4.9 min, concluding the method at 25 min. To evaluate the stability of the machine, a mixture containing six distinct authentic standards at varying concentrations (chlorogenic acid, daidzin, and naringenin at 1 ng/µL; phenylalanine at 2 ng/µL; and gallic acid, quercetin, and dihydroartemisinic acid at 3 ng/µL) was analyzed at the beginning, in the middle, and at the end of the experiment. For metabolite identification, a further data-dependent analysis was performed by performing a FAST-DDA in negative ionization mode.

The metabolites were putatively identified using the accurate mass (derived from the *m*/*z* ratio), retention time, and fragmentation pattern (MS/MS data from FAST-DDA analysis) that were compared with a proprietary database of authentic reference compounds, an in silico library of plant metabolites, and the relevant literature. Public databases, such as MoNA (https://mona.fiehnlab.ucdavis.edu/, accessed on 18 October 2023) and MassBank (https://massbank.eu/MassBank/Search, accessed on 18 October 2023), were also consulted. Positive ionization mode analysis was conducted exclusively to confirm the molecular ions observed in negative ionization mode. The metabolite abundance was obtained using Masslynx 4.1 (Waters).

### 2.5. Cytotoxicity Determination and Dose Selection

In this study, CHO-K1 cells were utilized to investigate cytotoxicity and genotoxicity. These cells are commonly used in such research due to their rapid growth and stable karyotype [19,20]. Previous studies have demonstrated that CHO-K1 cells exhibit a 79% sensitivity to known carcinogenic substances [21]. The CHO-K1 cells (603480) were obtained from CLS Cell Lines Service GmbH (Eppelheim, Germany). The methanolic extract of *S. pinnata* leaves was prepared by dissolving it in DMSO to create a stock solution of 50 mg/mL. To improve solubility, the extract was sonicated and stored at 4 °C until further use. CHO-K1 cells were cultured in Ham’s F12 medium, supplemented with 10% fetal bovine serum (FBS), 1% penicillin/streptomycin, and 1% glutamine. The cytotoxicity of *S. pinnata* extract on CHO-K1 cells was assessed using the MTT assay. Briefly, cells were seeded into 96-well plates (Primo^®^ Multiwall plates 96 flat bottoms, ET3096, Euroclone S.p.A., Pero, Italy) at a density of 3000 cells per well and incubated for 24 h at 37 °C in a 5% CO_2_ atmosphere. Following this, the cells were treated with various concentrations of *S. pinnata* extract, ranging from 0 to 1000 μg/mL in 2-fold increments, alongside a DMSO control (0.5%) and negative controls (cell medium) for an additional 24 h. After incubation, a 5 mg/mL MTT solution was introduced, and the cells were incubated for another 4 h at 37 °C in a 5% CO_2_ environment. Subsequently, the reaction solution was discarded, and 150 µL of DMSO (100%) was added to dissolve the formazan crystals. Absorbance was measured at 570 and 690 nm using a microplate reader (Synergy), and the cell viability percentage was calculated using the following formula as outlined in Equation (2):

(2) $$\%\;Viability\;of\;CHO-K1\;cells=\frac{Mean\;absorbance\;of\;the\;sample}{Mean\;absorbance\;of\;the\;control}\times 100$$

### 2.6. Genotoxicity Study Using CBMN Assay

#### 2.6.1. Genotoxic and Antigenotoxic Study

The in vitro micronucleus (MN) test was performed using the CBMN assay as reported [22] and following OECD guidelines [23,24]. The extracts of *S. pinnata* and MMC were both dissolved in DMSO, maintaining a final DMSO concentration of no more than 0.5%. CHO-K1 cells were seeded at a density of 3000 cells per well in a 96-well plate and were incubated in a humidified environment at 37 °C with 5% CO_2_ for 24 h before the introduction of the test samples. Following this incubation period, CHO-KI cells were treated with a negative control (DMOS at 0.5%) MMC at two concentrations of 0.025 and 0.50 µg/mL and *S. pinnata* extract at three concentrations of 75, 150, and 300 µg/mL, both in the presence and absence of MMC at 0.025 µg/mL, to evaluate the extract’s potential to mitigate the cytotoxic and genotoxic effects induced by MMC on CHO-K1 cells. After 24 h, the media was replaced, and the cells were treated with media containing 3 µg/mL of the cytokinesis-blocking agent Cytochalasin B (Cyto B) for another 24 h. The medium was then removed, and the cells were fixed with formaldehyde at a final concentration of 4% for 15 min. After discarding the fixation solution, the cells were washed twice with phosphate-buffered saline (PBS) for 3 min each. Following the washes, the PBS was removed, and the cells were stained with 100 µL per well of diluted bisbenzimide dye (Hoechst 33342 Staining Dye Solution) for 30 min in the dark at room temperature. The concentration range of *S. pinnata* extract (75, 150, and 300 µg/mL) was determined based on MTT assay results, which showed that the highest concentration caused cytotoxicity of less than 55 ± 5%, in accordance with OECD Guidelines. Concentrations above 300 µg/mL were found to be toxic to CHO-K1 cells, with cytotoxicity exceeding 50%. Therefore, 300 µg/mL was selected as the maximum concentration for assessing genotoxicity and antigenotoxicity. A concentration of 75 µg/mL was identified as non-toxic to CHO-K1 cells and was chosen as the minimum concentration for the study. Each concentration was tested in triplicate, with over 1000 binucleated cells scored for each concentration in every run. The experiment was repeated three times.

#### 2.6.2. Cytotoxicity Evaluation Under CBMN Assay

In vitro tests frequently fail to identify genotoxic substances unless the tested amounts cause some level of cytotoxicity. Excessive cytotoxicity, on the other hand, may produce false-positive results and cause incorrect data interpretation. Therefore, at the maximum concentration tested, it is advised that the cytotoxicity level not exceed 55 ± 5% [23,24]. Considering these factors, it is recommended to assess a minimum of three different concentrations of the test substance in the CBMN assay [24]. To address this issue in our experiments, we evaluated cytotoxicity as an integral part of the CBMN procedure, using the same cells that were utilized for counting micronuclei for the tested three concentrations used for the CBMN assay and applying two methods to assess the cytotoxicity under the CBNM assay as reported recently [22]. The first method involved calculating the percentage of cytotoxicity, which was derived from estimating the decrease in cell count following treatment, as outlined in Equation (3):

(3) $$\%\;Cytotoxicity=\frac{100\cdot [(number\;of\;cells\;before\;treatment)-(number\;of\;cells\;after\;treatment)}{number\;of\;untreated\;cells}$$

Another method utilizes CBPI, which reflects the average number of cell cycles that take place during exposure to cytochalasin B. This index is associated with a decreased ratio of binucleated to mononucleated cells. The percentage of cytotoxicity was calculated according to the formulas provided in Equations (4) and (5).

(4) $$CBPI=100\cdot \frac{\left[N_{1}+2\times N_{2}+3\times N_{3}\right]\;}{total\;number\;of\;cells}$$

(5) $$\%\;Cytotoxicity\left(CBPI\right)=100-\left[100\times \frac{CBPI\;of\;treated\;cells-1}{CBPI\;of\;untreated\;cells-1}\right]$$

N1 = number of mononucleated cells, N2 = number of binucleated cells, and N3 = number of multinucleated cells.

#### 2.6.3. Fluorescence Microscopic Image for MN Detection

Images were acquired using an inverted Leica DMi8 widefield microscope equipped with a Leica DFC9000GT CMOS camera and a 40× dry objective (Leica HC PL Fluotar L 40×/0.60), driven by LasX software (https://www.leica-microsystems.com/products/microscope-software/p/leica-las-x-ls/downloads/, accessed on 20 January 2025). The DAPI signal was recorded in 64 fields, uniformly distributed over the well area, for each condition, and each *S. pinnata* concentration was repeated in three wells so that at least 1000 binucleated cells per condition were analyzed. Each image was serially numbered and saved in the LAS X Office.

#### 2.6.4. Scoring of Binucleated Cells and Micronuclei

An accelerated and more accurate assessment of the micronucleus assay can be accomplished by automating the analysis of micronuclei using image processing software. In our study, binucleated cells and micronuclei were automatically identified using CellProfiler software (version 4.2.6). CellProfiler is an open-source, modular application for image analysis, specifically designed to manage extensive volumes of images. It provides a flexible platform for image analysis experts to collaborate, experiment, and develop innovative methodologies [25]. The software operates on a pipeline framework, comprising individual modules that process images in diverse manners, organized sequentially to create a thorough workflow. Initially, images captured using the widefield microscope were enhanced with the Leica Lightning deconvolution tool to improve the signal-to-noise ratio, facilitating the identification of nuclei and micronuclei by CellProfiler. Following this, images from the same experimental conditions were analyzed simultaneously by CellProfiler, as recently reported [22].

### 2.7. Statistical Analysis

Statistical analysis was performed using GraphPad Prism v.7. The number of independent experiments and details on statistical comparisons and levels of significance can be found in the captions of the figures.

## 3. Results

### 3.1. Metabolomic Profile of Specialized Metabolites of S. pinnata Leaf Extract

**Figure 1:**
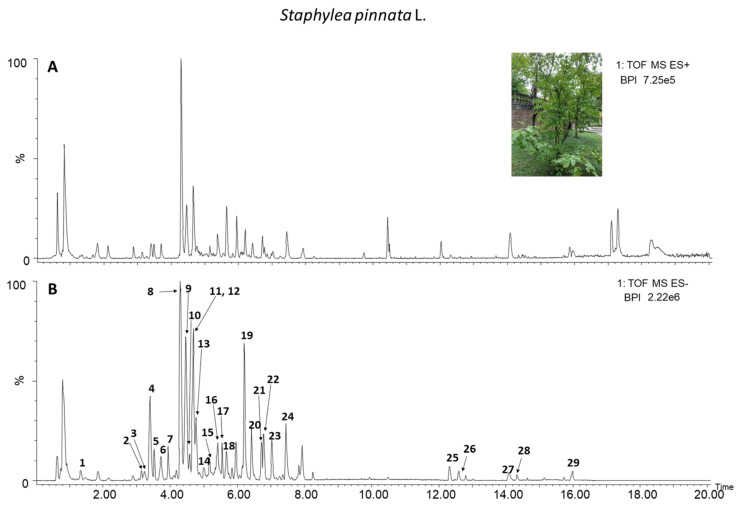
*S. pinnata* L. methanolic extract chromatograms. (**A**) Positive ionization mode; (**B**) negative ionization mode. The chromatograms show the main metabolites in *S. pinnata* methanol extracts.

An untargeted metabolomics approach was applied to analyze the leaf extract of *S. pinnata* using UPLC-HRMS. The chromatographic profiles of the samples, acquired in both negative and positive ionization modes, are presented in Figure 1. The numbers corresponding to the metabolites tentatively identified are shown in Table 1 and further details are reported in Supplementary Table S1.

As shown in Table 1, the highest value peak areas of the presented metabolites were for the procyanidin dimer 1, procyanidin trimer 3, procyanidin dimer 2, catechin, quercetin-o-rutinoside, and procyanidin tetramer 1, with peak areas of 84,368.54 ± 22,545.16, 76,013.76 ± 19,848.71, 40,872.21 ± 11,866.67, 32,270.00 ± 7888.78, and 28,883.55 ± 7503.36, respectively.

The phytochemical profile of *S. pinnata* leaf was strongly characterized by different B-type procyanidins and prodelphinidins. As shown in Figure 1, in the negative ionization mode, we identified three B-type procyanidins dimers (*m*/*z* 577.1346), four B-type procyanidins trimers (*m*/*z* 865.1980), one tetramer (*m*/*z* 1153.2614), and one B-type procyanidin pentamer (*m*/*z* 720.1582, double charges). The two prodelphinidins found in the extracts were a dimer (*m*/*z* 593.1295) and a trimer (*m*/*z* 881.1929). We also detected the presence of different O-glycosylated forms of two flavonols, quercetin and (iso)rhamnetin. The ending part of the chromatogram, where the chromatographic gradient reaches the highest acetonitrile levels, was characterized by the presence of different compounds belonging to the triterpenoid class. This group usually includes highly hydrophobic metabolites, mainly due to their chemical structure and elemental composition. In detail, we identified two isomers of maslinic acid (*m*/*z* 471.3474), asiatic acid and formic acid adducts (533.3478), and an oleanolic acid (*m*/*z* 455.3525). Other compounds putatively identified in *S. pinnata* extracts were citric acid, a dimeric lignin compound, piscidic acid ((p-Hydroxybenzyl) tartaric acid), a dihydroxybenzoic acid derivative, a benzyl alcohol-O-hexoside-pentoside, and esterification of caffeic acid with malic acid. Also, five unidentified compounds were observed. Information on the key characteristics of each numbered peak, including retention time, proposed annotation, experimental *m*/*z*, molecular formula, main adducts in both negative and positive acquisition modes, mass error in negative mode, and observed fragments in ms/ms are shown in Supplementary Table S1.

### 3.2. Cytotoxicity Evaluation of S. pinnata Extract

**Figure 2:**
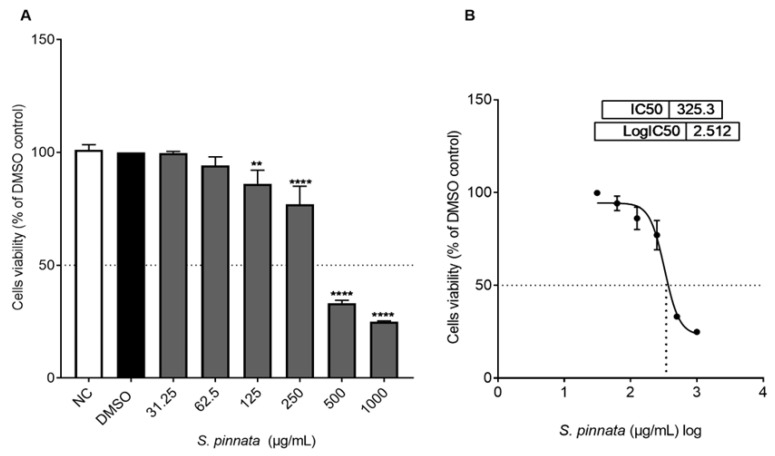
(**A**) The impact of different concentrations of *S. pinnata* extract (ranging from 0 to 1000 µg/mL) on the viability of CHO-K1 cells (% relative to DMSO control) after a 24 h treatment using the MTT assay. The figures illustrate the mean ± standard deviation derived from three independent experiments. Different symbols show statistically significant differences from the DMSO control (*p* < 0.0001). (**B**) A nonlinear regression analysis of log-transformed concentration values (curve fitting) was utilized to ascertain the IC_50_ value. Statistical evaluations were conducted using GraphPad Prism Version 7, employing ANOVA followed by the Tukey multiple comparison post-test (** *p* = 0.0027, **** *p* < 0.0001).

Cytotoxicity is defined as the capacity of a substance to induce cell death [26]. To assess the cytotoxic impact of crude *S. pinnata* methanolic extract on CHO-K1 cells, the MTT cell viability assay was performed, with results illustrated in (Figure 2A,B). CHO-K1 cells incubated with *S. pinnata* extract for 24 h at concentrations below 125 µg/mL did not demonstrate significant cytotoxic effects when compared to both negative control (NC) and DMSO control cells. In contrast, at concentrations of 125, 250, 500, and 1000 µg/mL, a significant reduction in the viability of CHO-K1 cells was observed in comparison to the DMSO control cells. At the highest concentration tested, 1000 µg/mL, cell viability was reduced to 25% relative to both NCs and DMSO controls. The concentration of *S. pinnata* extract could not be increased beyond 1000 µg/mL due to its low solubility and its precipitation with the media. The extract exhibited an IC50 value of 325 µg/mL.

### 3.3. Genotoxicity Assessment Using CBMN Assay

#### 3.3.1. Cytotoxicity and Binucleated Cells Evaluation

**Figure 3:**
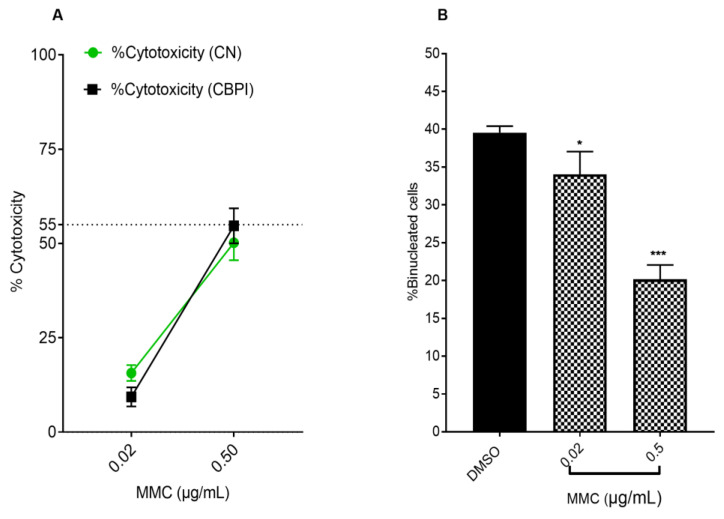
(**A**) The Cytotoxic effect of MMC on CHO-K1 cells [cytotoxicity (%) CN (green) and CBPI (black)] after treatment for 24 h, and additionally cells were incubated with Cyto B for another 24 h. (**B**) % of binucleated cells in CHO-K1 cells after 24 h incubation with DMSO control and different concentrations of MMC. The figures illustrate the mean ± standard deviation derived from three independent experiments. Different symbols show statistically significant differences from the NC using ANOVA followed by Tukey’s multiple comparisons test in GraphPad Prism, with comparisons made to the NC group. (* *p* = 0.0439, *** *p* < 0.001).

In our investigation, three different concentrations of *S. pinnata* extracts were utilized: 75, 150, and 300 μg/mL. This selection was based on the results of the MTT assay, which indicated an IC_50_ value of 325 µg/mL. Notably, the highest concentration of 300 μg/mL exhibited cytotoxicity levels below 55 ± 5% when compared to untreated control cells (DMSO control cells). To determine the genotoxic potential of *S. pinnata* extract, the initial focus was on assessing cytotoxicity. This evaluation was conducted as part of the MN experiment, utilizing the same cell lines for scoring the MN. Our results demonstrated that MMC, a well-established genotoxic clastogenic agent, led to an increase in cytotoxicity percentages, measured as % cytotoxicity CN, in a dose-dependent manner. Specifically, the percentages recorded were 15.67 ± 2.08% and 50.21 ± 4.65% at concentrations of 0.025 and 0.5 μg/mL, respectively, in comparison to NC cells (cells treated with DMSO at 0.4%) (Figure 3A).

With the CBPI method, MMC caused a significant increase in CBPI cytotoxicity percentages, ranging from 9.33 ± 2.51% to 54.69 ± 4.64% at concentrations of 0.025 and 0.5 μg/mL, respectively, compared to the NC cells (Figure 3A). To inhibit cytokinesis and promote the formation of binucleated post-mitotic cells, Cyto B was used to block actin assembly. In the NC group, CHO-K1 cells treated with 3 μg/mL of Cyto B for 24 h showed a mean binucleated cell percentage of 40.75 ± 2.29% (Figure 3B). In contrast, MMC-treated cells exhibited a significant reduction in binucleated cell formation, with percentages of 35.37 ± 1.53% and 20.16 ± 1.89% at MMC concentrations of 0.025 and 0.5 μg/mL, respectively, compared to the NC cells. Therefore, the MMC concentration of 0.025 μg/mL was selected for further experiments to assess the genotoxicity and antigenotoxicity of *S. pinnata* extracts, as it displayed lower cytotoxicity compared to the MMC at 0.5 μg/mL (Figure 3B).

**Figure 4:**
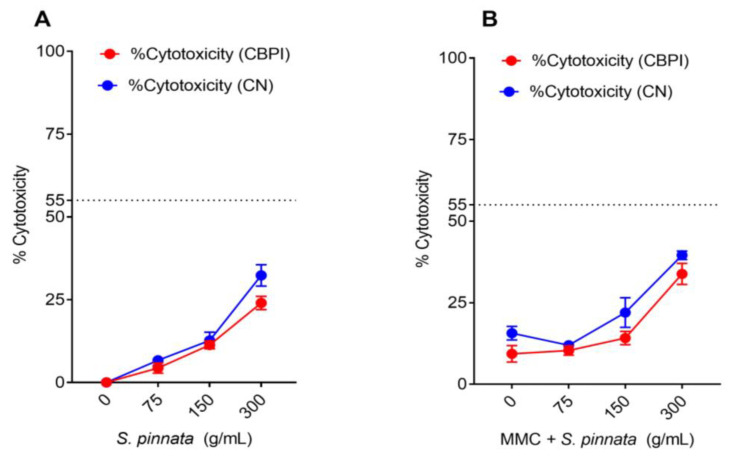
(**A**,**B**) The cytotoxic effect of *S. pinnata* extracts on CHO-K1 cells [cytotoxicity (%) CN (blue) and CBPI (red)] with or without 0.025 μg/mL of MMC for 24 h, with cells additionally incubated with Cyto B for another 24 h. Data were collected from three independent experiments.

The concentrations of *S. pinnata* applied, specifically 75, 150, and 300 μg/mL, resulted in cytotoxicity levels of 6.67 ± 1.53%, 12.67 ± 2.52%, and 32.33 ± 3.22%, respectively, as measured by the %CN method (Figure 4A). Conversely, the cytotoxicity assessed using the CBPI method indicated lower levels of cytotoxicity at 4.33 ± 1.53%, 11.33 ± 1.16%, and 24.15 ± 2.02% for the same concentrations of *S. pinnata* when compared to the NC cells (Figure 4A). When cells were treated with *S. pinnata* at a concentration of 75 μg/mL in the presence of MMC, a slight reduction in cytotoxicity was observed, yielding a value of 11.94 ± 1.0% as opposed to 15.65 ± 2.08% for MMC alone. Notably, an increase in cytotoxicity was recorded for cells treated with *S. pinnata* at concentrations of 150 and 300 μg/mL, showing values of 21.97 ± 4.55% and 39.54 ± 1.29%, respectively, in comparison to MMC-treated cells, which exhibited 15.67 ± 2.08% (Figure 4B). This indicates a significant cytotoxic effect of *S. pinnata* at higher concentrations, particularly at 300 μg/mL. In the presence of MMC, the cytotoxicity measured by CBPI revealed values of 10.34 ± 1.39%, 14.17 ± 2.05%, and 33.28 ± 3.23% for cells treated with MMC and *S. pinnata* at 75, 150, and 300 μg/mL, respectively, when compared to MMC-treated cells that showed 9.33 ± 2.52% (Figure 4B).

**Figure 5:**
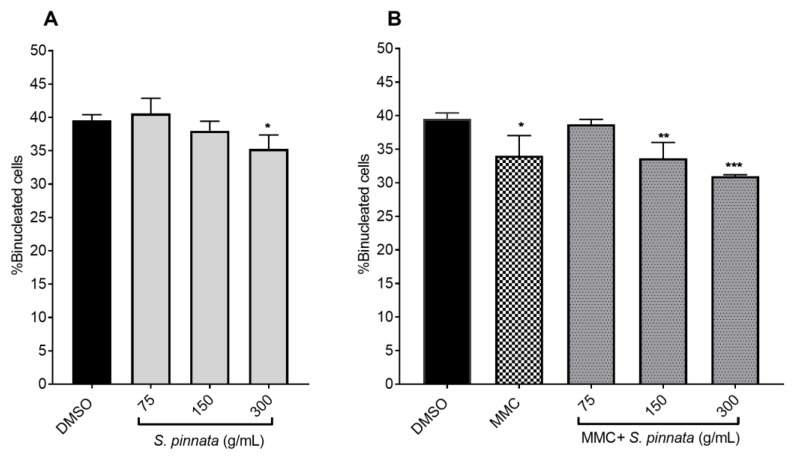
Formation of binucleated cells (%) in CHO-K1 cells treated with *S. pinnata* extract at three different concentrations without (**A**) or with (**B**) MMC at 0.025 μg/mL for 24 h and the cells subsequently incubated with 3 μg/mL Cyto B for 24 h. Data were collected from three independent experiments. Different symbols show statistically significant differences from the NC using ANOVA followed by Tukey’s multiple comparisons test in GraphPad Prism, with comparisons made to the NC group. (* *p* = 0.0118, ** *p* = 0.0078, *** *p* = 0.0006).

No notable differences in the formation of binucleated cells were observed when treated with *S. pinnata* extracts at concentrations of 75 and 150 μg/mL compared to NC cells. However, at a concentration of 300 μg/mL, the formation of binucleated cells was significantly reduced compared to the NC cells. Binucleated cells formed were 41 ± 2.92%, 38 ± 1.44%, and 35 ± 2.10% for *S. pinnata* treated cells at 75, 150, and 300 μg/mL, respectively, while the NC exhibited a percentage of 40 ± 1.00% (Figure 5A).

Conversely, when cells were treated with *S. pinnata* extract alongside MMC, the percentage of binucleated cells was significantly lower in those treated with *S. pinnata* at concentrations of 150 and 300 μg/mL compared to the negative control group. The percentages of binucleated cells formed were 39 ± 1.01%, 34 ± 2.36%, and 30 ± 0.2% for *S. pinnata* treated cells at 75, 150, and 300 μg/mL, respectively, compared to the NC cells, which had a percentage of 40 ± 1.00% (Figure 5B).

#### 3.3.2. Micronuclei Analysis

**Figure 6:**
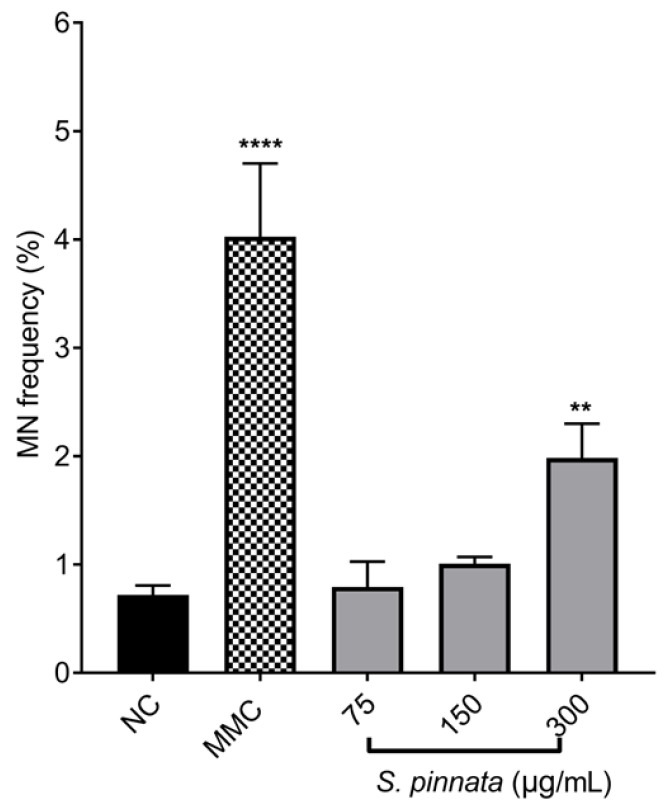
The effect of different *S. pinnata* extract treatments on the MN frequency (%) in CHO-K1 cells. Cells were treated for 24 h with 3 different concentrations of *S. pinnata* extract, then followed by 24 h incubation with 3 μg/mL of Cyto B. Graphs represent data collected from three independent experiments. One-way ANOVA and Tukey’s multiple comparisons test using GraphPad Prism 7 software, were applied to calculate statistical significance in comparison with NC. (** *p* = 0.0046, **** *p* < 0.0001). MN frequency (%) was calculated from the following equation: micronuclei frequency (%) = binucleated cells with MN /binucleated cells *100.

In our findings, the application of the MMC at a concentration of 0.025 μg/mL for 24 h on CHO-K1 cells led to a notable rise in the frequency of binucleated cells with micronuclei (MN frequency), measuring 4.15 ± 0.68%, compared to 0.72 ± 0.09% observed in NC cells (Figure 6). This concentration served as a positive control in this study, with the percentage of binucleated cells being close to that of NC cells.

**Figure 7:**
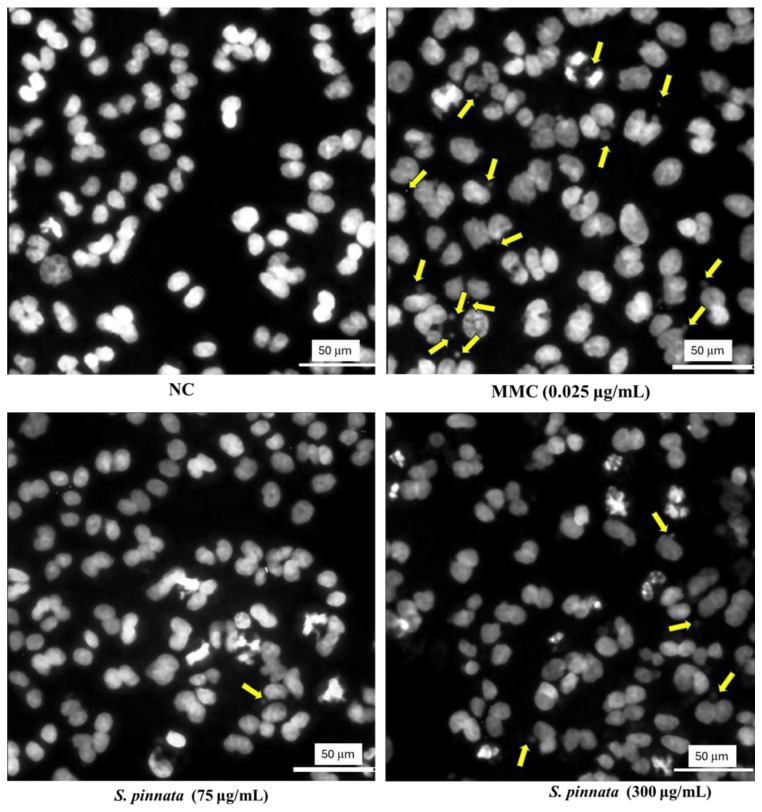
Microscopic images illustrating the micronuclei formation in CHO-K1 binucleated cells, observed with a 40× objective after a 24 h incubation period without treatment (NC) or treated with 0.025 μg/mL MMC or *S. pinnata* extract at 75 and 300 μg/mL. The DNA of CHO-K1 cells was stained using Hoechst dye. Yellow arrows indicate the micronuclei and the white line with a value of 50 µm in the image represents the scale bar.

Upon treatment with *S. pinnata* at concentrations of 75 and 150 μg/mL, no significant difference in the MN frequency was observed compared to NC cells (Figure 6). The MN frequency remained at 0.79 ± 0.2% and 1.01 ± 0.06% for 75 and 150 μg/mL *S. pinnata* treatments, respectively, in comparison to the NC value of 0.72 ± 0.09%. However, cells treated with *S. pinnata* at a concentration of 300 μg/mL showed a significant increase in MN frequency (1.99 ± 0.32%) compared to NC (0.72 ± 0.09%), indicating the genotoxic potential of *S. pinnata* extract treatment at the highest concentration and under the experimental conditions used. However, the MN frequency of *S. pinnata* extract (1.99 ± 0.32%) at 300 μg/mL was significantly lower than the MN frequency of MMC at 0.025 μg/mL (4.03 ± 0.68%), suggesting a weak genotoxic effect. Therefore, our study demonstrates a relationship between the induction of cytotoxicity and genotoxicity caused by *S. pinnata* extract at a concentration of 300 μg/mL. Images depicting the formation of micronuclei in binucleated CHO-K1 cells following a 24 h incubation with MMC at a concentration of 0.025 μg/mL and *S. pinnata* at the tested concentrations are presented in Figure 7.

#### 3.3.3. Antigenotoxicity Determination

**Figure 8:**
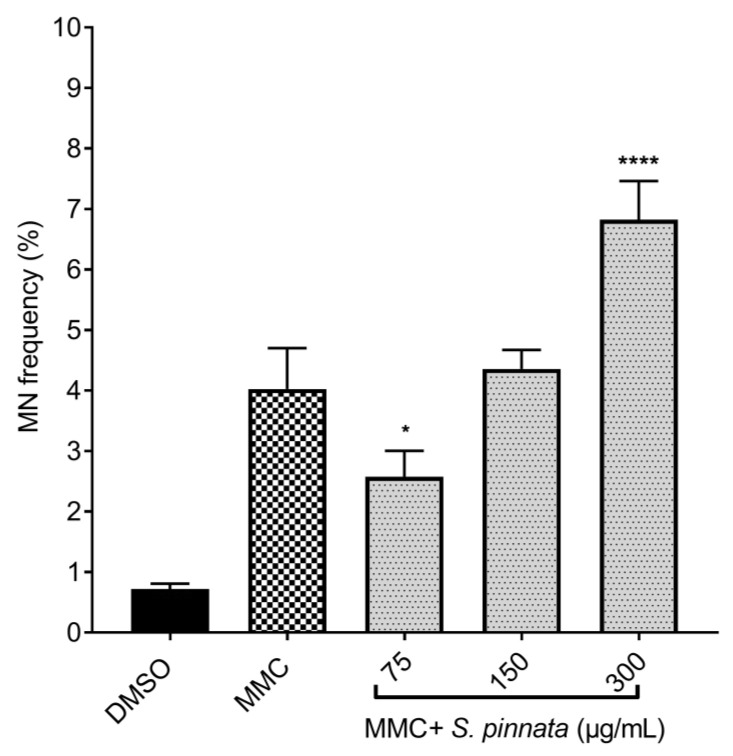
MN frequency (%) in CHO-K1 cells treated with MMC at 0.02 μg/mL alone or MMC + *S. pinnata* extract at three different concentrations of 57, 150, and 300 μg/mL for 24 h, followed by incubation with Cyto B at 3 μg/mL for another 24 h. The graphs display data from three independent experiments. Statistical significance was determined using one-way ANOVA followed by Tukey’s multiple comparisons test, conducted with GraphPad Prism 7 software, comparing the results to the MMC control (* *p* = 0.0135, **** *p* < 0.0001).

In a separate phase of the experiment, cells were treated with *S. pinnata* extract in combination with MMC. The MMC concentration of 0.025 μg/mL resulted in a significant increase in the MN frequency compared to the NC cells under the experimental conditions. The results showed a substantial reduction in MN frequency in cells treated with *S. pinnata* extract at a lower concentration of 75 μg/mL alongside MMC (2.58 ± 0.43%) when compared to cells treated with MMC alone (4.03 ± 0.68%) (Figure 8). In contrast, no significant differences in MN frequency were observed in cells treated with *S. pinnata* extract at 150 μg/mL in the presence of MMC (4.36 ± 0.32%) compared to those treated with MMC alone (4.03 ± 0.68%) (Figure 8).

**Figure 9:**
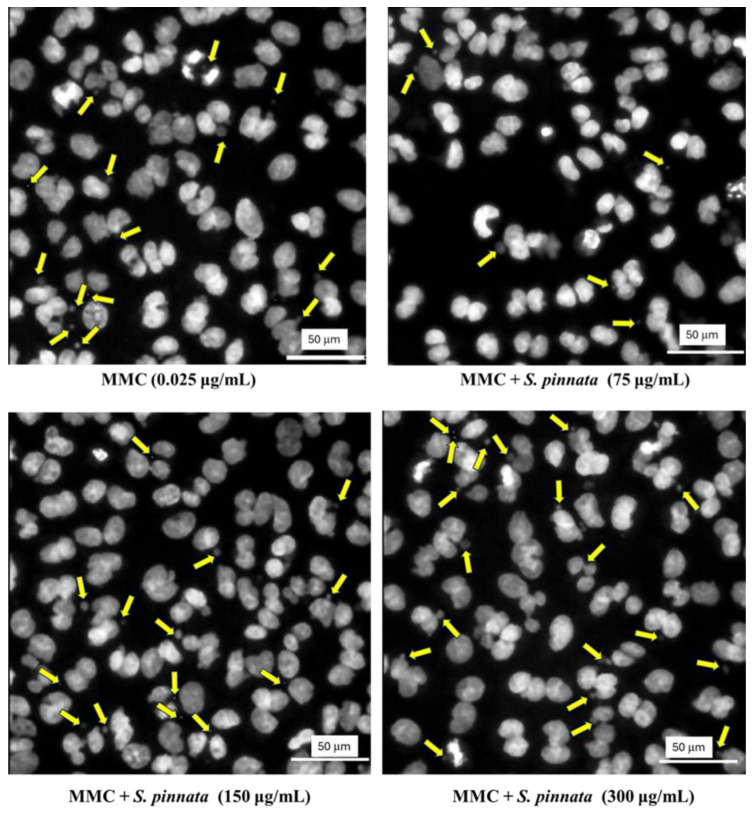
Representative microscopic images of MN formation in binucleated CHO-K1 cells with 40× objectives after 24 h incubation with 0.025 μg/mL MMC alone or MMC + *S. pinnata* extract at 75, 150, and 300 μg/mL. CHO-K1 cell DNA was stained with Hoechst dye. Yellow arrows indicate micronuclei and the white line with a value of 50 µm in the image represents the scale bar.

Notably, cells treated with *S. pinnata* at 300 μg/mL in combination with MMC displayed a significant increase in MN frequency (6.83 ± 0.64%) compared to those treated with MMC alone (4.03 ± 0.68%) (Figure 8), suggesting that the *S. pinnata* extract enhances MMC-induced genotoxicity at this highest concentration. As a result, a correlation between the induction of both cytotoxicity and genotoxicity was observed in cells treated with *S. pinnata* extract at 300 μg/mL. Representative images of MN formation in binucleated CHO-K1 cells treated with *S. pinnata* extract and MMC at 0.025 μg/mL are presented in Figure 9.

## 4. Discussion

While the safety and effectiveness of many plants utilized as traditional medicines for human consumption have not been comprehensively studied, individuals continue to rely on them due to their accessibility. To ensure the safety of these traditional medicinal plants, it is crucial to assess their potential cytotoxic and genotoxic effects, as this evaluation is a key factor in determining the efficacy of these remedies. Genotoxicity testing in vitro has gained increasing importance as a method for evaluating the safety of different drugs and chemicals. The formation of micronuclei acts as a biomarker for assessing the DNA-damaging potential of the substances under investigation. In this regard, the CBMN assay has become a widely used tool for detecting genotoxicity and identifying compounds that may reduce genotoxic and cytotoxic effects [27].

The biological activities exhibited by plant extracts, which consist of a variety of natural compounds, result from the unique roles of individual components and their interactions. In toxicological research, the chemical characterization of these extracts is essential, as it establishes a foundation for understanding the interactions among various compounds within biological systems. By determining the chemical properties and structures of a compound, researchers can evaluate its potential toxicity, which may include cytotoxic, genotoxic, and antigenotoxic effects. Therefore, this study aimed to characterize the metabolomic profile of the methanolic extract obtained from *S. pinnata* leaves using UPLC-HRMS and to examine its cytotoxic, genotoxic, and antigenotoxic effects on CHO-K1 cells through the CBMN assay.

The metabolomic investigation of *S. pinnata* indicated that the most abundant compounds are proanthocyanidins or condensed tannins, including various B-type procyanidins and prodelphinidins. Additionally, O-glycosylated variants of two flavonols, namely quercetin and (iso)rhamnetin, were also detected. Numerous tannins are acknowledged for their protective characteristics, which include anti-inflammatory, anti-fibrotic, anti-microbial, and anti-diabetic properties [28]. Procyanidins are generally accumulated in flowers, fruits, bark, and the seeds of various plants [29,30] and may exert significant health-promoting effects, such as antioxidant [31], antibacterial [32], anti-inflammatory [33], antineoplastic anti-allergic [34,35], lipid-lowering, and anti-obesity effects [36]. Because of these properties, they are widely recognized and employed in the healthcare industry [37].

In our initial experiments, we evaluated the cytotoxic effects of *S. pinnata* leaf extract at concentrations varying from 0 to 1000 μg/mL on CHO-K1 cells utilizing the MTT assay. The existing literature on the in vitro cytotoxicity of *S. pinnata* is limited, with a notable study by [8] demonstrating that water infusions of *S. pinnata* leaves administered to A431 cells yielded ED_50_ values of 35.3 ± 3.8 and 12.6 ± 1.9 μg/mL after 24 and 72 h of incubation, respectively, as assessed by the MTT assay. The cytotoxic effects observed were linked to the presence of various phytochemicals, including flavonoids, polyphenol triterpenes, and steroids [8]. Additionally, the crude hydroalcoholic extract of *S. pinnata* aerial parts, along with its fractions obtained through liquid–liquid extraction (LLE) and solid-phase extraction (SPE), were evaluated for their cytotoxicity against HeLa cells using the MTT assay. The findings indicated that the crude hydroalcoholic extract of *S. pinnata* at a concentration of 500 μg/mL for 24 h did not exhibit cytotoxicity towards HeLa and HaCaT cells. Conversely, both fractions of *S. pinnata* demonstrated significant toxicity against both cell lines at the same concentration [1]. As a result, the IC_50_ value obtained from this extract aligns with the range reported for other *S. pinnata* extracts in the literature.

The genotoxic effect of *S. pinnata* extract in the presence of MMC was evaluated. To our knowledge, this study is the first to explore the genotoxic and antigenotoxic characteristics of the methanolic extract derived from *S. pinnata* leaves. There is limited research on the genotoxic and antigenotoxic effects of *Staphylea* species, making it challenging to compare our findings with those of other *Staphylea* species. MMC has been recognized as genotoxic in both in vitro and in vivo testing involving mammalian cells and animal models, and it is widely acknowledged as a carcinogenic substance [38]. In our study, MMC was utilized as a positive control, validating the sensitivity of the CBMN assay and demonstrating a clear positive response at the concentration used. According to OECD guidelines, a definitive positive result for the substance tested is indicated when at least one concentration shows a statistically significant increase compared to the concurrent negative control under any experimental conditions. Consequently, our findings indicate that the methanolic leaf extract of *S. pinnata* exhibits genotoxic effects in CHO-K1 cells at the highest concentration tested, as confirmed by the in vitro CBMN assay.

It was noted that the extract from *S. pinnata* leaves exhibited contrasting effects that were dependent on the concentration applied, demonstrating a hormetic concentration-dependent response, characterized by the most pronounced effect observed at 300 μg/mL. Hormesis, as previously defined, refers to a phenomenon where a stressor agent yields beneficial effects at lower doses while producing adverse effects at higher doses, thus exhibiting varying effects based on the concentration tested [39]. Similar concentration-dependent antigenotoxic hormetic effects have been observed in various plant extracts. For instance, the stem extract of *Dendrobium speciosum* demonstrated protective properties against DNA damage induced by 4NQO in HePG2 cells at lower concentrations, as evaluated through the comet assay [40]. Furthermore, the lowest concentration of wastewater extracts from olive mill demonstrated an antigenotoxic hormetic effect when tested on HepaRG™ cells [41]. In addition, extract from *Gentiana lutea* at a lower tested dose reduced the genotoxicity induced by 2-amino-3-methyl-3H-imidazo[4,5-f]quinolone and 2-amino-1-methyl-6-phenylimidazo[4,5-b]pyridine (PhIP) in genetically modified Salmonella typhimurium TA1535, [42].

Genotoxicity investigations involving various plant extracts indicate that the observed genotoxic effects are contingent upon employed concentrations. For instance, *Hemidesmus indicus* (L.) R.Br. root ethanolic extract exhibited genotoxic effects at higher concentrations while demonstrating antigenotoxic properties at lower concentrations [43]. Conversely, both oil and water extracts from *Eugenia uniflora* L. showed mutagenic effects at lower concentrations but were classified as antimutagenic at elevated concentrations [44]. Moreover, manool extracted from the dried leaves of *Salvia officinal* L. was genotoxic at high employed doses, yet it conferred a protective effect against chromosome damage induced by methyl methanesulphonate at lower doses used [45]. Additionally, certain plant extracts, including *Artemisia vulgaris* L. and *Artemisia alba* Turra., displayed both genotoxic and antigenotoxic phytochemical effects at the same concentrations tested [46]. This phenomenon can be explained by the presence of complex mixtures of phytochemicals within the plant extracts, which may interact synergistically, while some phytochemicals may exhibit genotoxicity and others may mitigate these effects.

Plant extracts consist of a variety of metabolomic compounds, and their diverse biological activities arise from the distinct functions of individual components or their interactions [47]. Many studies have shown that various phenolic compounds display antioxidant or pro-oxidant properties that depend on their concentration [48]. Consequently, compounds may exhibit genotoxic or antigenotoxic protective effects when presented alone or in combination [46]. In our investigation, the genotoxic effect observed in treated cells with the highest concentration of *S. pinnata* may be attributed to the presence of certain specialized metabolites in the phytocomplex. Our results revealed the presence of O-glycosylated forms of two flavonols, namely quercetin and (iso)rhamnetin. Previous studies have shown that quercetin and its natural glycosides may exert various mutagenic and genotoxic effects in vitro [17,49] and in vivo [50].

The antigenotoxic effect observed for the extract of *S. pinnata* at its lowest concentration might be attributed to proanthocyanidins. In particular, the B-type procyanidins and prodelphinidines, as shown in Table 1, are metabolites reported to exert antioxidant activity, activate antioxidant cell machinery, and form complexes with other macromolecules. Previous studies, indeed, reported that proanthocyanidin-rich extracts derived from grape seeds did not exhibit any genotoxic effects in vivo [51,52] and significantly reduced DNA damages induced by H_2_O_2_ in Fao cells, as evidenced by the Comet assay [53]. Furthermore, proanthocyanidin-rich extracts from grape seeds resulted in a notable reduction in the frequency of micronuclei in vivo [19]. A rich fraction of proanthocyanidin obtained from the stem bark of *Stryphnodendron adstringens* was found to be non-genotoxic and showed an antigenotoxic effect against damages caused by cyclophosphamide [54]. Procyanidins from grape seeds activate antioxidative enzymes and the glutathione cycle in both in vivo and in vitro studies [55,56], thus contributing to the enhancement of cellular antioxidant capacity. Finally, the capacity of tannins to form complexes with various macromolecules might also contribute to protective activity through its antioxidants effect, as suggested in previous studies [57].

## 5. Conclusions

This study provides important insights into the metabolomic profile and potential cytotoxic, genotoxic, and antigenotoxic effects of the *S. pinnata* leaf extract belonging to Italian flora. Our findings show that the methanolic extract of *S. pinnata* leaf contains a wide variety of metabolites from several chemical classes, with the most abundant metabolites being different B-type procyanidins and prodelphinidins in addition to quercetin and (iso)rhamnetin (Table 1). To the best of our knowledge, this study is the first to investigate the genotoxic and antigenotoxic properties of the methanolic extract from *S. pinnata* leaves in vitro. Under the experimental conditions used in this study, our results indicate that the genotoxic effect of *S. pinnata* leaf extract is influenced by the concentration used. *S. pinnata* leaf methanolic extract demonstrates cytotoxic properties and shows both genotoxic and antigenotoxic effects. The levels of genotoxicity and cytotoxicity of *S. pinnata* extract were found to increase with higher concentrations used. The genotoxic and antigenotoxic effects of *S. pinnata* leaf extract are suggested to be linked to the phytochemical presence in the extracts, such as procyanidins and prodelphinidins, in addition to quercetin and (iso)rhamnetin derivatives as shown in Table 1. This study utilized crude extracts of *S. pinnata* leaves, with complex mixtures of biologically active compounds. Some of these compounds may exhibit cytotoxic and/or genotoxic effects, while others may possess cytoprotective and/or antigenotoxic properties. Consequently, this study indicates that despite the natural origin of the *S. pinnata* leaf extract, its indiscriminate use could lead to genotoxic and toxic effects, particularly at elevated concentrations. Therefore, careful consideration of dosage is essential for its safe consumption. Further investigations are necessary to assess the safety of *S. pinnata* leaf extracts using animal models and to elucidate the mechanisms underlying these effects.

## Figures and Tables

**Table 1:** Abundances (peak area—AU: arbitrary units).

Peak Number	Compound Name	Pool 1	Pool 2	Pool 3	Mean	STD
1	Citric acid	511.00	1116.00	574.00	733.67	297.49
2	Dimeric lignin (coniferyl alcohol derivative)	2428.54	1725.64	2077.01	2077.07	314.35
3	Prodelphinidin trimer 1	6983.25	7073.67	3991.68	6016.20	1568.71
4	Procyanidin trimer 1	32,232.76	28,374.76	16,314.96	25,640.83	7426.97
5	Piscidic acid	1922.00	3984.00	1991.00	2632.33	1047.45
6	Prodelphinidin dimer 1	9874.15	10,441.22	5545.04	8620.14	2395.42
7	Dihydroxybenzoic acid derivative	5822.43	4847.15	5390.92	5353.50	437.12
8	Procyanidin dimer 1	105,135.88	91,645.37	56,324.36	84,368.54	22,545.16
9	Procyanidin dimer 2	50,754.83	46,068.60	25,793.22	40,872.21	11,866.67
10	Procyanidin trimer 2	3683.00	3535.00	1618.00	2945.33	1030.28
11	Catechin	38,654.00	35,950.00	22,206.00	32,270.00	7888.78
12	Procyanidin trimer 3	91,430.89	86,030.83	50,579.56	76,013.76	19,848.71
13	Procyanidin tetramer 1	34,292.34	33,138.63	19,219.69	28,883.55	7503.36
14	Procyanidin pentamer 1	26,054.97	25,956.82	14,713.18	22,241.66	5831.70
15	Benzyl alcohol-O-hexoside-pentoside	6665.07	6029.68	6905.57	6533.44	404.76
16	Procyanidin trimer 4	22,770.24	21,524.11	12,379.30	18,891.22	5074.80
17	Caffeoyl malic acid	153.00	121.00	68.00	114.00	38.40
18	Procyanidin dimer 3	10,106.00	8839.00	4776.00	7907.00	2490.57
19	Quercetin-O-rutinoside	38,144.35	30,459.67	29,199.06	32,601.03	4330.69
20	Quercetin-O-hexoside	15,264.34	13,449.42	11,052.62	13,255.46	1889.52
21	Quercetin-O-malonylhexoside	11,796.04	9409.35	9075.77	10,093.72	1327.02
22	Isorhamnetin-O-rutinoside	18,728.16	16,116.04	15,764.19	16,869.47	1448.31
23	Isorhamnetin-O-hexoside	13,694.87	12,686.62	9526.32	11,969.27	1945.28
24	Isorhamnetin-O-malonylhexoside	22,373.07	18,442.05	16,459.41	19,091.51	2692.09
25	Asiatic acid	5178.89	4480.20	4386.19	4681.76	387.36
26	Asiatic acid isomer	4763.01	4240.51	4323.26	4442.26	251.19
27	Maslinic acid	21,692.20	20,709.16	20,638.08	21,013.14	526.95
28	Maslinic acid isomer	12,830.20	12,069.47	12,129.90	12,343.19	378.21
29	Oleanolic acid	2769.13	2637.53	2565.13	2657.26	92.50

## Data Availability

Data are contained within the article or Supplementary Materials. The original contributions presented in this study are included in the article/Supplementary Materials. Further inquiries can be directed at the corresponding author.

## Supplementary Materials

The following supporting information can be downloaded at: https://www.mdpi.com/article/10.3390/biom15030385/s1, Table S1: Putative identification of the metabolites presented in *S. pinnata* leaf methanol extracts.

## References

[1] Paolillo I., Roscigno G., Innangi M., Zorrilla J.G., Petraglia G., Russo M.T., Carraturo F., Guida M., Pollice A., Cimmino A. (2024). Health-Promoting Properties of Natural Flavonol Glycosides Isolated from *Staphylea pinnata* L.. Int. J. Mol. Sci..

[2] Madeja J., Harmata K., Kołaczek P., Karpińska-Kołaczek M., Piątek K., Naks P., Morel J.-P., Mercuri A.M. (2009). Bracken (*Pteridium aquilinum* (L.) Kuhn), mistletoe (*Viscum album* (L.)) and bladder-nut (*Staphylea pinnata* (L.))-mysterious plants with unusual applications. Culturaland ethnobotanical studies. Plants and Culture: Seeds of the Cultural Heritage of Europe.

[3] Heiss A.G., FilipoviĆ D., Nedelcheva A., Ruß-Popa G., Wanninger K., Schramayr G., Perego R., Jacomet S. (2014). A Fistful of Bladdernuts: The Shifting Uses of *Staphylea pinnata* L. as Documented by Archaeology, History, and Ethnology. Folk Life.

[4] Redžić S., Ferrier J., Pieroni A., Quave C. (2014). The Use of Wild Plants for Human Nutrition During a War: Eastern Bosnia (Western Balkans). Ethnobotany and Biocultural Diversities in the Balkans.

[5] Sircelj H., Vidrih R., Veberič R., Mikulič-Petkovšek M. (2019). Evaluation of bioactive constituents in European bladdernut (*Staphylea pinnata* L.) seed kernels. J. Food Compos. Anal..

[6] Maia A. (2020). Medicinal Remedies is Plant are Species of *Staphylea pinnata* L. and Family *Staphyleaceae* Lindl. in the Georgia (South Caucasus). Agric. Res. Technol..

[7] Lacikova L., Muselik J., Masterova I., Grancai D. (2007). Antioxidant activity and total phenols in different extracts of four *Staphylea* L. Species. Molecules.

[8] Lacikova L., Jancova M., Muselik J., Masterova I., Grancai D., Fickova M. (2009). Antiproliferative, cytotoxic, antioxidant activity and polyphenols contents in leaves of four *Staphylea* L. species. Molecules.

[9] Rutkowska J., Pasqualone A. (2025). Plant Extracts as Functional Food Ingredients. Foods.

[10] Al-Naqeb G., Kalmpourtzidou A., Giampieri F., De Giuseppe R., Cena H. (2024). Genotoxic and antigenotoxic medicinal plant extracts and their main phytochemicals: “A review”. Front. Pharmacol..

[11] De Quadros A.P.O., Oshiiwa B., Petreanu M., Niero R., Rosa P.C.P., Sawaya A.C.H.F., Mantovani M.S., O’Neill De Mascarenhas Gaivão I., Maistro E.L. (2023). *Rubus rosifolius* (Rosaceae) stem extract induces cell injury and apoptosis in human hepatoma cell line. Toxicol In Vitro.

[12] Quadros Gomes A.R., da Rocha Galucio N.C., de Albuquerque K.C.O., Brígido H.P.C., Varela E.L.P., Castro A.L.G., Vale V.V., Bahia M.O., Rodriguez Burbano R.M., de Molfeta F.A. (2021). Toxicity evaluation of *Eleutherine plicata* Herb. extracts and possible cell death mechanism. Toxicol. Rep..

[13] Jantová S., Nagy M., Ruzeková L., Grancai D. (2000). Antibacterial activity of plant extracts from the families Fabaceae, Oleaceae, Philadelphaceae, Rosaceae and Staphyleaceae. Phytother. Res..

[14] Kim M., Jee S.C., Shin M.K., Han D.H., Bu K.B., Lee S.C., Jang B.Y., Sung J.S. (2022). Quercetin and Isorhamnetin Reduce Benzo[a]pyrene-Induced Genotoxicity by Inducing RAD51 Expression through Downregulation of miR-34a. Int. J. Mol. Sci..

[15] Musonda C.A., Chipman J.K. (1998). Quercetin inhibits hydrogen peroxide (H2O2)-induced NF-kappaB DNA binding activity and DNA damage in HepG2 cells. Carcinogenesis.

[16] Undeğer U., Aydin S., Başaran A.A., Başaran N. (2004). The modulating effects of quercetin and rutin on the mitomycin C induced DNA damage. Toxicol. Lett..

[17] Engen A., Maeda J., Wozniak D.E., Brents C.A., Bell J.J., Uesaka M., Aizawa Y., Kato T.A. (2015). Induction of cytotoxic and genotoxic responses by natural and novel quercetin glycosides. Mutat. Res. Genet. Toxicol. Environ. Mutagen..

[18] Zorzi G., Gambini S., Negri S., Guzzo F., Commisso M. (2023). Untargeted Metabolomics Analysis of the Orchid Species *Oncidium sotoanum* Reveals the Presence of Rare Bioactive C-Diglycosylated Chrysin Derivatives. Plants.

[19] Santos G.S., Tsutsumi S., Vieira D.P., Bartolini P., Okazaki K. (2014). Effect of Brazilian propolis (AF-08) on genotoxicity, cytotoxicity and clonogenic death of Chinese hamster ovary (CHO-K1) cells irradiated with ^60^Co gamma-radiation. Mutat. Res. Genet. Toxicol. Environ. Mutagen..

[20] El Hosry L., Di Giorgio C., Birer C., Habib J., Tueni M., Bun S.S., Herbette G., De Meo M., Ollivier E., Elias R. (2014). In vitro cytotoxic and anticlastogenic activities of saxifragifolin B and cyclamin isolated from *Cyclamen persicum* and *Cyclamen libanoticum*. Pharm. Biol..

[21] Kirkland D., Aardema M., Henderson L., Müller L. (2005). Evaluation of the ability of a battery of three in vitro genotoxicity tests to discriminate rodent carcinogens and non-carcinogens I. Sensitivity, specificity and relative predictivity. Mutat. Res..

[22] Al-Naqeb G., Zorzi G., Oldani A., Azzalin A., Avesani L., Guzzo F., Pascale A., De Giuseppe R., Cena H. (2024). Phytochemical Profile and In Vitro Cytotoxic, Genotoxic, and Antigenotoxic Evaluation of *Cistus monspeliensis* L. Leaf Extract. Int. J. Mol. Sci..

[23] Organization for Economic Co-operation and Development (OECD) (2023). Test No. 487: In Vitro Mammalian Cell Micronucleus Test. OECD Guidelines for the Testing of Chemicals.

[24] Phelps J.B., Garriott M.L., Hoffman W.P. (2002). A protocol for the in vitro micronucleus test. II. Contributions to the validation of a protocol suitable for regulatory submissions from an examination of 10 chemicals with different mechanisms of action and different levels of activity. Mutat. Res..

[25] Ramadhani D., Purnami S. (2013). Automated Detection of Binucleated Cell and Micronuclei using CellProfiler 2.0 Software. Hayati J. Biosci..

[26] Salunke M., Wakure B., Wakte P. (2022). HR-LCMS assisted phytochemical screening and an assessment of anticancer activity of Sargassum Squarrossum and Dictyota Dichotoma using in vitro and molecular docking approaches. J. Mol. Struct..

[27] Hashimoto K., Nakajima Y., Matsumura S., Chatani F. (2011). Comparison of four different treatment conditions of extended ex-posure in the in vitro micronucleus assay using TK6 lymphoblastoid cells. Regul. Toxicol. Pharmacol..

[28] Rajasekar N., Sivanantham A., Ravikumar V., Rajasekaran S. (2021). An overview on the role of plant-derived tannins for the treatment of lung cancer. Phytochemistry.

[29] Li W., He Y., Zhao H., Peng L., Li J., Rui R., Ju S. (2021). Grape Seed Proanthocyanidin Ameliorates FB_1_-Induced Meiotic Defects in Porcine Oocytes. Toxins.

[30] Enomoto T., Nagasako-Akazome Y., Kanda T., Ikeda M., Dake Y. (2006). Clinical effects of apple polyphenols on persistent allergic rhinitis: A randomized double-blind placebo-controlled parallel arm study. Investig. Allergol. Clin. Immunol..

[31] Grace M.H., Xiong J., Esposito D., Ehlenfeldt M., Lila M.A. (2019). Simultaneous LC-MS quantification of anthocyanins and non-anthocyanin phenolics from blueberries with widely divergent profiles and biological activities. Food. Chem..

[32] Nawrot-Hadzik I., Matkowski A., Hadzik J., Dobrowolska-Czopor B., Olchowy C., Dominiak M., Kubasiewicz-Ross P. (2021). Proanthocyanidins and Flavan-3-Ols in the Prevention and Treatment of Periodontitis-Antibacterial Effects. Nutrients.

[33] Tian Y., Yang C., Yao Q., Qian L., Liu J., Xie X., Ma W., Nie X., Lai B., Xiao L. (2019). Procyanidin B2 Activates PPARγ to Induce M2 Polarization in Mouse Macrophages. Front. Immunol..

[34] Choy Y.Y., Fraga M., Mackenzie G.G., Waterhouse A.L., Cremonini E., Oteiza P.I. (2016). The PI3K/Akt pathway is involved in procyanidin-mediated suppression of human colorectal cancer cell growth. Mol. Carcinog..

[35] Sazwi N.N., Nalina T., Abdul Rahim Z.H. (2013). Antioxidant and cytoprotective activities of *Piper betle*, *Areca catechu*, *Uncaria gambir* and betel quid with and without calcium hydroxide. BMC Complement. Altern. Med..

[36] Wang Q.Q., Gao H., Yuan R., Han S., Li X.X., Tang M., Dong B., Li J.X., Zhao L.C., Feng J. (2020). Procyanidin A2, a polyphenolic compound, exerts anti-inflammatory and anti-oxidative activity in lipopolysaccharide-stimulated RAW264.7 cells. PLoS ONE.

[37] Lee Y.A., Cho E.J., Tanaka T., Yokozawa T. (2007). Inhibitory activities of proanthocyanidins from persimmon against oxidative stress and digestive enzymes related to diabetes. J. Nutr. Sci. Vitaminol..

[38] Tang J., Li B., Hong S., Liu C., Min J., Hu M., Li Y., Liu Y., Hong L. (2017). Punicalagin suppresses the proliferation and invasion of cervical cancer cells through inhibition of the β-catenin pathway. Mol. Med. Rep..

[39] Carneiro C.C., da Costa Santos S., de Souza Lino R., Bara M.T., Chaibub B.A., de Melo Reis P.R., Chaves D.A., da Silva A.J., Silva L.S., de Melo e Silva D. (2016). Chemopreventive effect and angiogenic activity of punicalagin isolated from leaves of *Lafoensia pacari* A. St.-Hil. Toxicol. Appl. Pharmacol..

[40] Moretti M., Cossignani L., Messina F., Dominici L., Villarini M., Curini M., Marcotullio M.C. (2013). Antigenotoxic effect, composition and antioxidant activity of *Dendrobium speciosum*. Food Chem..

[41] Rondini T., Branciari R., Franceschini E., Acito M., Fatigoni C., Roila R., Ranucci D., Villarini M., Galarini R., Moretti M. (2024). Olive Mill Wastewater Extract: In Vitro Genotoxicity/Antigenotoxicity Assessment on HepaRG Cells. Int. J. Environ. Res. Public Health.

[42] Cvetković S., Todorović S., Nastasijević B., Mitić-Ćulafić D., Đukanović S., Knežević-Vukčević J., Nikolić B. (2020). Assessment of genoprotective effects of *Gentiana lutea* extracts prepared from plants grown in field and in vitro. Ind. Crops Prod..

[43] Ananthi R., Chandra N., Santhiya S.T., Ramesh A. (2010). Genotoxic and antigenotoxic effects of *Hemidesmus indicus* R. Br. root extract in cultured lymphocytes. J. Ethnopharmacol..

[44] Kuhn A.W., Tedesco M., Laughinghouse H.D., Flores F.C., Silva C.D., Canto-Dorow T.S., Tedesco S.B. (2015). Mutagenic and antimutagenic effects of *Eugenia uniflora* L. by the *Allium cepa* L. test. Caryologia.

[45] Nicolella H.D., de Oliveira P.F., Munari C.C., Costa G.F., Moreira M.R., Veneziani R.C., Tavares D.C. (2014). Differential effect of manool a diterpene from *Salvia officinalis*, on genotoxicity induced by methyl methanesulfonate in V79 and HepG2 cells. Food Chem. Toxicol..

[46] Jakovljević M.R., Grujičić D., Vukajlović J.T., Markovic A., Milutinović M.G., Stanković M.S., Vukovic N.L., Vukić M.D., Milošević-Djordjević O. (2020). In vitro study of genotoxic and cytotoxic activities of methanol extracts of *Artemisia vulgaris* L. and *Artemisia alba* Turra. S. Afr. J. Bot..

[47] Vaou N., Stavropoulou E., Voidarou C.C., Tsakris Z., Rozos G., Tsigalou C., Bezirtzoglou E. (2022). Interactions between Medical Plant-Derived Bioactive Compounds: Focus on Antimicrobial Combination Effects. Antibiotics.

[48] Azqueta A., Collins A. (2016). Polyphenols and DNA Damage: A Mixed Blessing. Nutrients.

[49] Utesch D., Feige K., Dasenbrock J., Broschard T.H., Harwood M., Danielewska-Nikiel B., Lines T.C. (2008). Evaluation of the potential in vivo genotoxicity of quercetin. Mutat. Res..

[50] Hobbs C.A., Koyanagi M., Swartz C., Davis J., Kasamoto S., Maronpot R., Recio L., Hayashi S.M. (2018). Comprehensive evaluation of the flavonol anti-oxidants, alpha-glycosyl isoquercitrin and isoquercitrin, for genotoxic potential. Food Chem. Toxicol..

[51] Yamakoshi J., Saito M., Kataoka S., Kikuchi M. (2002). Safety evaluation of proanthocyanidin-rich extract from grape seeds. Food Chem. Toxicol..

[52] Erexson G.L. (2003). Lack of in vivo clastogenic activity of grape seed and grape skin extracts in a mouse micronucleus assay. Food Chem. Toxicol..

[53] Llópiz N., Puiggròs F., Céspedes E., Arola L., Ardévol A., Bladé C., Salvadó M.J. (2004). Antigenotoxic effect of grape seed procyanidin extract in Fao cells submitted to oxidative stress. J. Agric. Food Chem..

[54] Costa M.A., Ishida K., Kaplum V., Koslyk E.D., de Mello J.C., Ueda-Nakamura T., Dias Filho B.P., Nakamura C.V. (2010). Safety evaluation of proanthocyanidin polymer-rich fraction obtained from stem bark of *Stryphnodendron adstringens* (BARBATIMAO) for use as a pharmacological agent. Regul. Toxicol. Pharmacol..

[55] Roig R., Cascón E., Arola L., Bladé C., Salvadó M.J. (1999). Moderate wine consumption protects the rat against oxidation in vivo. Life Sci..

[56] Roig R., Cascón E., Arola L., Bladé C., Salvadó M.J. (2002). Procyanidins protect Fao cells against hydrogen peroxide-induced oxidative stress. Biochim. Biophys. Acta..

[57] Haslam E. (1996). Natural polyphenols (vegetable tannins) as drugs: Possible modes of action. J. Nat. Prod..

